# Efficacy and Safety of Xialiqi for the Treatment of Benign Prostatic Hyperplasia in a Randomized Trial

**DOI:** 10.1016/j.euros.2025.10.006

**Published:** 2025-10-25

**Authors:** Feiya Yang, Chao Yang, Xin Wang, Dong Chen, Zhong Wang, Jianwen Wang, Chaozhao Liang, Lin Hua, Hao Ping, Jianxin Lu, Zhiping Wang, Wei Li, Nianzeng Xing

**Affiliations:** a Department of Urology, National Cancer Center, Beijing, China; b Department of Urology, RenJi Hospital, Shanghai Jiao Tong University, Shanghai, China; c Clinical Trials Center, National Cancer Center, Beijing, China; d Clinical Trials Center, Shanxi Province Cancer Hospital/Shanxi Hospital, Taiyuan, China; e Shanghai Ninth People’s Hospital, Shanghai JiaoTong University, Shanghai, China; f Beijing Chao-Yang Hospital, Capital Medical University, Beijing, China; g First Affiliated Hospital of Anhui Medical University, Hefei, China; h School of Biomedical Engineering, Capital Medical University, Beijing, China; i Beijing Tongren Hospital, Capital Medical University, Beijing, China; j Guang’anmen Hospital, China Academy of Chinese Medical Sciences, Beijing, China; k Second Hospital of Lanzhou University, Lanzhou, China; l Second Hospital of Hebei Medical University, Shijiazhuang, China

**Keywords:** Xialiqi, Lower urinary tract symptoms, Benign prostatic hyperplasia, International Prostate Symptom Score, Traditional Chinese medicine

## Abstract

**Background and objective:**

Benign prostatic hyperplasia (BPH) is a major cause of lower urinary tract symptoms (LUTS) in men aged ≥50 yr. Xialiqi capsules are a traditional Chinese medicine mainly used for the treatment of moderate BPH-related LUTS.

**Methods:**

We conducted a multicenter, randomized, double-blind, placebo-controlled clinical trial of Xialiqi treatment for 8 wk in a cohort of 395 patients. Changes in BPH-related LUTS were assessed in terms of the International Prostate Symptom Score (IPSS). Irritative and obstructive IPSS subscores and secondary efficacy indicators, including quality of life (QoL) and International Index of Erectile Function (IIEF-5) scores, were also assessed. Safety and tolerability were assessed in terms of adverse events (AEs) and serious AE.

**Key findings and limitations:**

At the final time point of 8 wk, the least-squares treatment difference in IPSS between the Xialiqi and placebo groups was −2.33 (95% confidence interval −3.01 to −1.64). Analysis of covariance results suggested that there was no significant difference between the groups in terms of interactions of subgroups or between parameter subgroups. Results for secondary efficacy measures, including Chronic Prostatitis Symptom Index and QoL scores, revealed greater improvement with Xialiqi than with placebo. Safety results show that Xialiqi was well tolerated and AEs were mild. Limitations include the lack of long-term follow-up.

**Conclusions and clinical implications:**

Xialiqi was associated with an improvement in LUTS over an 8-wk period among men with moderate BPH symptoms.

**Patient summary:**

We looked at the safety and efficacy of Xialiqi capsules for the treatment of benign enlargement of the prostate gland in a large Chinese population. We found that Xialiqi provides an early improvement in urinary symptoms and prostate enlargement in men with moderate symptoms relating to an enlarged prostate.

## Take Home Message

Our results show that Xialiqi capsules were safe and effective for the treatment of benign prostatic hyperplasia (BPH) in a Chinese cohort. Xialiqi provide early improvements in lower urinary tract symptoms and prostatic enlargement in men with moderate BPH.

## 1 Introduction

Benign prostatic hyperplasia (BPH) is one of the most common chronic diseases in the male population and is the major cause of lower urinary tract symptoms (LUTS) among men aged ≥50 yr. Drug administration is still the main treatment strategy for patients with mild to moderate BPH, with α-receptor blockers and 5α-reductase inhibitors (5-ARIs), alone or combined, the main drug classes prescribed [1]. These two therapeutic classes are effective for the treatment of mild to moderate LUTS associated with BPH.

Drug treatment in this setting has focused mainly on the static or dynamic component of BPH. For the static component, 5-ARIs including finasteride and dutasteride can inhibit the proliferative action of androgens, and these drugs account for approximately 23% of the worldwide prostate pharmacotherapy market [2]. 5-ARIs for BPH treatment usually take effect slowly, and up to 6 mo may elapse before effective relief of symptoms. Side effects include impotence, a decrease in libido, and abnormal ejaculation [[3], [4], [5]]. α-Adrenoceptor antagonists are effective for the treatment of BPH [6], and tamsulosin and tamoxifen account for approximately 65% of global prostate drugs [2]. α-Adrenoceptor antagonists can relieve urethral obstruction and LUTS by reducing matrix smooth-muscle tension [7]. However, vasodilation is a side effect of α_1_-adrenoceptor antagonists, and selective blocking of α_1_-adrenoceptor subtypes is related to some abnormal ejaculation effects [8].

Some traditional Chinese medicines have shown positive effects in the treatment of BPH. Xialiqi capsules are a traditional Chinese medicine mainly used for the treatment of mild to moderate BPH. The main capsule ingredients are *Astragalus*, glossy privet fruit, talc, selfheal, lychee seed, amber, cinnamon, and amur corktree bark, and Xialiqi capsules strengthen the spleen and kidney, remove water, and disperse knots. Research has revealed that Xialiqi capsules have good diuretic, proliferative, anti-inflammatory, and analgesic effects in animal models of BPH, chronic bacterial prostatitis, and chronic aseptic prostatitis [9]. Previous studies have explored the effects of Xialiqi on the expression of PCNA, caspase-3, IL-8, TNF-α, dihydrotestosterone, SOD, and malondialdehyde in rat models of BPH [10]. These studies confirmed that Xialiqi can significantly reduce prostate wet weight and the prostate index in a rat model of BPH. The rationale for the efficacy of Xialiqi in BPH is described in the Supplementary material.

To further explore the safety and effectiveness of Xialiqi for BPH treatment, we conducted a multicenter, randomized, double-blind, placebo-controlled clinical trial. Safety and tolerability were assessed in terms of the incidence of adverse events (AEs) and serious AEs (SAEs).

## 2 Patients and methods

We conducted a multicenter, randomized, double-blind, placebo-controlled trial at 14 centers in China. The trial was registered in the Chinese Clinical Trial Registry (ChiCTR 1900022393). The inclusion criteria were as follows: (1) men with a diagnosis of BPH; (2) International Prostate Symptom Score (IPSS) between 8 and 19 points; (3) prostate volume ≥30 ml; (4) maximum urinary flow rate (Q_max_) <15 ml/s; (5) age 50–80 yr; and (6) voluntary participation in the study with a signed consent form. The exclusion criteria were: (1) postvoid residual urine volume (PVR) >150 ml; (2) prostate-specific antigen (PSA) >4 ng/ml; (3) serious hepatorenal dysfunction; (4) severe chronic obstructive pulmonary disease or respiratory failure; (5) severe infection; (6) neuropsychiatric disease; (7) malignant tumor; (8) allergy to any of the ingredients of the investigational drug; (9) participation in other clinical studies; and (10) presence of any disease other than BPH that can cause urination symptoms or changes in the urine flow rate according to the judgment of the researchers.

We used block randomization and double-blind methods to manage random assignment of participants to the treatment arms via an interactive web response system (IWRS). The randomization list was generated by the randomization statistician in the statistical unit using SAS software and imported into the IWRS. The block size was 4, and the allocation ratio between the arms was 1:1.

In the Xialiqi treatment arm, three Xialiqi capsules (0.45 g/capsule) were taken three times daily. The placebo was similar to Xialiqi in characteristics such as color, smell, taste, shape, and texture. The placebo and investigational drug were consistent in specifications, appearance, packaging, labeling, and markings, making it difficult for clinical trial participants to distinguish between them. Throughout the study, the patients, researchers, and study personnel were unaware of group assignments. Adherence to medication was evaluated via a complete record of drug distribution and recovery. Postbaseline study visits were conducted at 4 wk and 8 wk.

The primary efficacy measure was total IPSS. Secondary efficacy measures included prostate volume, Q_max_, average urine flow rate (Q_ave_), PVR, and International Index of Erectile Function (IIEF-5) score. For the safety analysis, all AEs reported by patients were evaluated. Laboratory values were measured at baseline and at the end of the study.

According to the study protocol, no imputation for missing data will be used for data that were missing completely at random or in relation to safety issues. For data either missing at random or missing not at random, multiple imputation will be used for imputation of missing data.

### 2.1 Sample size calculation

A total sample size of 312 participants (156 per group) is required to detect a mean difference of 1.3 points in IPSS between groups with 80% power at a two-sided significance level of 5%, assuming a common standard deviation of 4.1. This calculation is based on a two-sample t test for comparison of means, inflated to 196 per group for loss to follow-up and other reasons.

### 2.2 Calculation

Analysis of urodynamic measurements and efficacy variables was conducted using the per-protocol set (PPS) comprising patients who received one or more doses of double-blind treatment and had urodynamic measurement results at baseline and postbaseline. Analysis of safety variables was conducted using the safety analysis set comprising patients who received one or more doses of treatment. The treatment effect between the arms was estimated as the least-squares treatment difference (LSTD). Treatment effects in terms of total IPSS, obstructive IPSS, and irritative IPSS were estimated using analysis of covariance (ANCOVA) models that included treatment, hospital, and baseline parameters as variables. The changes in IPSS from baseline to study endpoint in different subgroups were also analyzed with ANCOVA model, with variables including treatment groups, hospitals, baseline parameters, subgroups, and treatment × subgroup interactions. Changes in secondary efficacy measures from baseline to the end of the study period were assessed in the two treatment arms via generalized estimating equation (GEE) models with time, treatment, hospital, baseline parameters, and treatment × time interactions as variables; time was treated as a fixed categorical effect.

## 3 Results

### 3.1 Study cohort and baseline characteristics

**Fig. 1:**
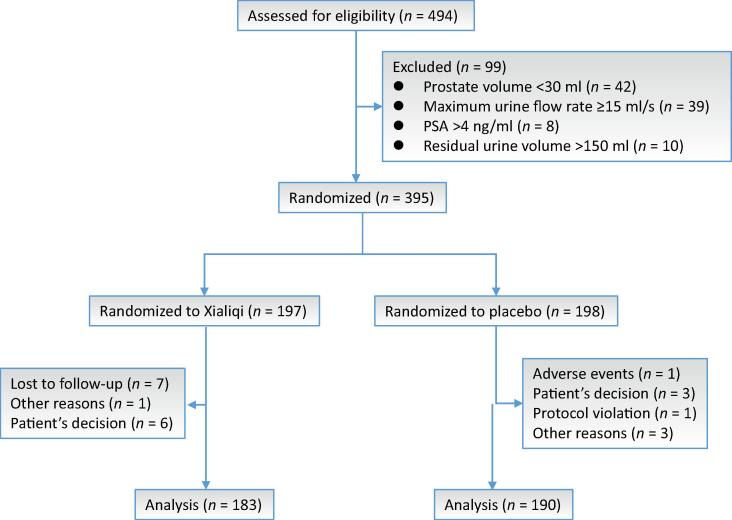
Study flow diagram. PSA = prostate-specific antigen.

From November 2019 to June 2022, 494 patients with BPH were considered for enrollment. After patients who did not meet the criteria were excluded, 395 patients were randomized, 197 to the Xialiqi arm and 198 to the placebo arm. Of these patients, 373/395 (94.4%) completed the trial; 15 men in the Xialiqi arm and seven in the placebo arm discontinued participation (Fig. 1).

Baseline characteristics did not differ significantly between the treatment arms (Table 1). In the Xialiqi arm, median age was 64 yr (interquartile range 58–68) and median body mass index was 24.99 kg/m^2^ (interquartile range 22.76–27.15). The mean IPSS at baseline was 14.22 (standard deviation 3.46) and mean prostate volume was 43.2 cm^3^ (standard deviation 15.54). There were no significant differences in baseline characteristics between the Xialiqi and placebo arms.

**Table 1:** Baseline characteristics of the patient population by treatment arm

Variable	Xialiqi (*n* = 183)	Placebo (*n* = 190)
Median age, yr (IQR)	64 (58–68)	63 (58–68)
Median body mass index, kg/m^2^ (IQR)	24.99 (22.76–27.15)	24.77 (23.03–26.88)
Mean total IPSS (SD)	14.22 (3.46)	14.08 (3.60)
Mean QoL score (SD)	3.9 (0.84)	3.83 (0.82)
Mean CPSI score (SD)	16.37 (5.76)	16.06 (5.15)
Mean average flow rate, ml/s (SD)	4.21 (1.83)	4.17 (1.82)
Mean maximum flow rate, ml/s (SD)	8.99 (3.17)	8.86 (3.20)
Mean QS (SD)	88.78 (7.90)	88.14 (7.71)
Mean prostate volume, cm^3^ (SD)	43.20 (15.54)	44.18 (16.67)
Median PSA, ng/ml (IQR)	1.54 (0.90–2.58)	1.64 (0.96–2.48)
Median IIEF-5 score (IQR)	5 (1–12)	3 (1–13)
Median PVR, cm^3^ (IQR)	13.0 (2.0–38.5)	15.0 (0.0–41.6)

CPSI = Chronic Prostatitis Symptom Index; IIEF = International Index of Erectile Function; IPSS = International Prostate Symptom Score; IQR = interquartile range; PSA = prostate-specific antigen; PVR = postvoid residual volume; QoL = quality of life; SD = standard deviation.

### 3.2 Primary efficacy measure

**Fig. 2:**
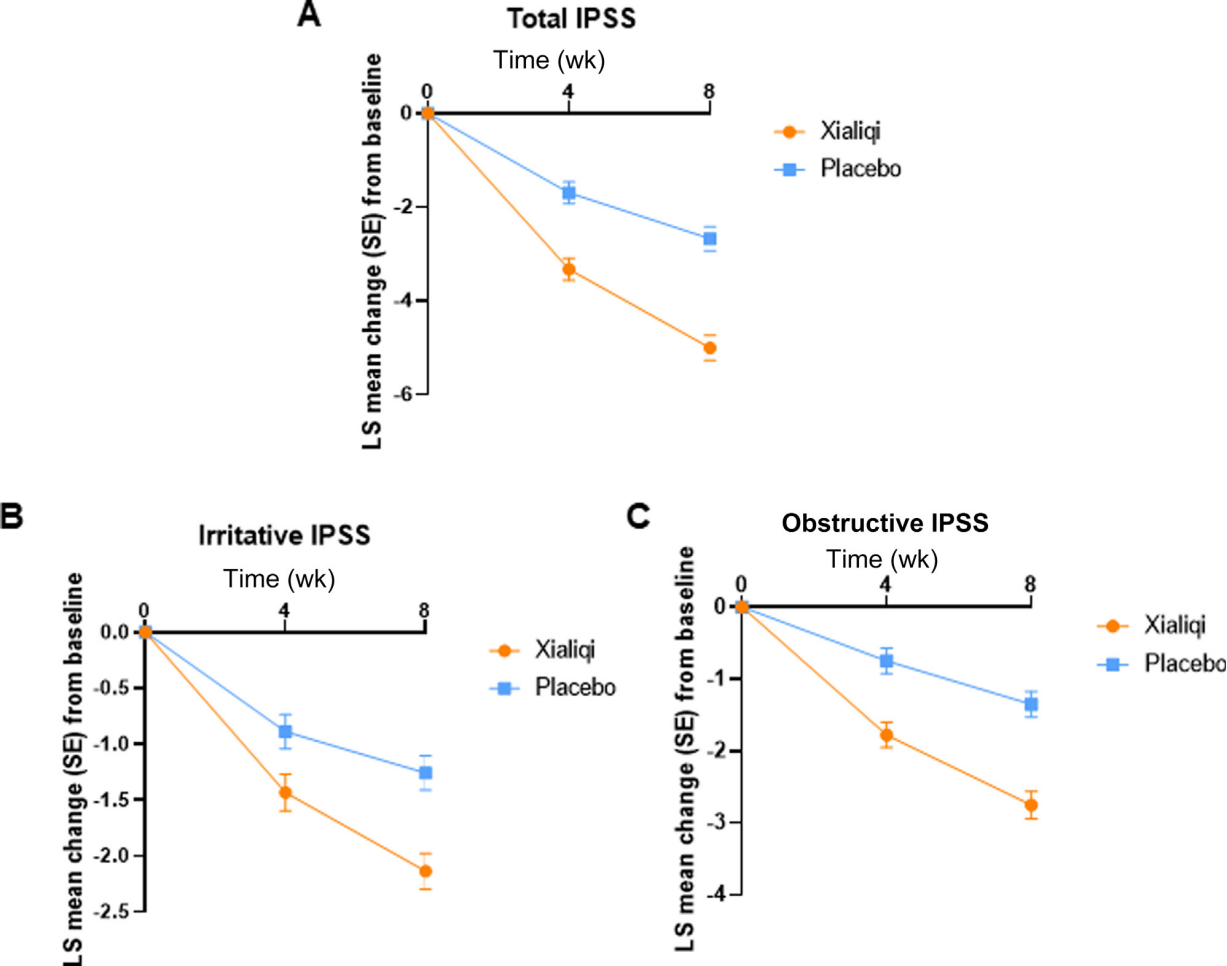
Changes in International Prostate Symptom Score (IPSS0 over time. Analysis of covariance used treatment, hospital, and baseline values as variables. Least-squares (LS) mean change in (A) total IPSS, (B) irritative IPSS, and (C) obstructive IPSS. SE = standard error.

The least squares (LS) method was used to assess changes in IPSS. The LS mean for total IPSS tended to decrease from baseline in both treatment arms (Fig. 2A). The LSTD for total IPSS between the Xialiqi and placebo arms was −1.63 (95% confidence interval [CI] −2.20 to −1.06; *p* < 0.001) at 4 wk, and −2.33 (95% CI −3.01 to −1.64; *p* < 0.001) at 8 wk (Table 2). The results indicate that the improvement in total IPSS was greater in the Xialiqi arm than in the placebo.

**Table 2:** Estimates of the treatment effect on total, obstructive, and irritative IPSS

	LS mean IPSS change from baseline (SE) a		LSTD (95% CI)	*p* value
	Xialiqi (*n* = 183)	Placebo (*n* = 190)		
**At 4 wk**				
Total IPSS	−3.33 (0.24)	−1.70 (0.23)	−1.63 (−2.20 to −1.06)	<0.001
Irritative IPSS	−1.43 (0.16)	−0.88 (0.15)	−0.55 (−1.06 to −0.04)	0.023
Obstructive IPSS	−1.78 (0.17)	−0.75 (0.18)	−1.03 (−1.60 to −0.46)	0.004
**At 8 wk**				
Total IPSS	−5.01 (0.27)	−2.68 (0.26)	−2.33 (−3.01 to −1.64)	<0.001
Irritative IPSS	−2.14 (0.16)	−1.26 (0.15)	−0.88 (−1.37 to −0.39)	0.006
Obstructive IPSS	−2.75 (0.19)	−1.35 (0.17)	−1.40 (−1.95 to −0.85)	<0.001

CI = confidence interval; CPSI = Chronic Prostatitis Symptom Index; IPSS = International Prostate Symptom Score; mLS = least-squares mean; LSTD = LS treatment difference (difference in mLS change from baseline between Xialiqi and placebo); SE = standard error.

a Analysis of covariance was performed using treatment group, hospital, and baseline values as variables.

LS mean results for changes in irritative IPSS (Fig. 2B) and obstructive IPSS (Fig. 2C) were also analyzed. The LSTD for irritative IPSS between the Xialiqi and placebo arms was −0.55 (95% CI −1.06 to −0.04; *p* = 0.023) at 4 wk, and −0.88 (95% CI −1.37 to −0.39; *p* = 0.006) at 8 wk (Table 2), indicating a better improvement with Xialiqi than with placebo. The LSTD for obstructive IPSS between the Xialiqi and placebo arms was −1.03 (95% CI −1.60 to −0.46; *p* = 0.004) at 4 wk, and −1.40 (95% CI −1.95 to −0.85; *p* < 0.001) at 8 wk (Table 2). The results show a greater improvement in obstructive IPSS with Xialiqi than with placebo. In conclusion, improvement in both irritative IPSS and obstructive IPSS were better with Xialiqi than with placebo.

### 3.3 IPSS changes across subgroups

Table 3 shows the changes in IPSS from baseline to 8 wk for subgroups stratified by treatment, baseline values, subgroups, and interaction subgroups according to ANCOVA models. The results indicate that there were no statistically significant differences between subgroups or the interactions of subgroups.

**Table 3:** Subgroup analysis results for the International Prostate Symptom Score change from baseline^a^

Variable	Xialiqi (*n* = 183)			Placebo (*n* = 190)			LSTD	*p*_group_	*p*_subgroup_	*p*_interaction_
	*n*	mLS	SE	n	mLS	SE	(95% CI)			
Age										
≥65 yr	98	−4.97	0.37	111	−2.67	0.38	−2.30 (−3.34 to −1.28) *	<0.001	0.84	0.99
≥65 yr	85	−5.05	0.34	79	−2.72	0.32	−2.33 (−3.24 to −1.40) *	<0.001		
BMI										
≥25 kg/m^2^	91	−5.11	0.35	104	−3.08	0.36	−2.03 (−2.98 to −1.01) *	<0.001	0.19	0.47
<25 kg/m^2^	92	−4.91	0.35	86	−2.37	0.33	−2.54 (−3.49 to −1.61) *	<0.001		
PSA										
<1.5 ng/ml	84	−5.09	0.37	84	−2.57	0.37	−2.52 (−3.72 to −1.67) *	<0.001	0.90	0.55
≥1.5 ng/ml	99	−4.92	0.34	106	−2.83	0.33	−2.09 (−3.06 to −0.94) *	<0.001		
IIEF-5 score										
<22 points	174	−4.93	0.35	180	−2.54	0.35	−2.39 (−3.09 to −1.69) *	<0.001	0.40	0.59
≥22 points	9	−4.84	1.22	10	−3.77	1.21	−1.07 (−4.39 to 2.27)	0.53		
CPSI category										
Mild	77	−4.79	0.45	75	−2.86	0.44	−1.93 (−3.00 to −0.87) *	<0.001	0.80	0.51
Moderate	102	−4.99	0.43	112	−2.49	0.42	−2.50 (−3.41 to −1.61) *	<0.001		
Severe	4	−6.88	1.80	3	−2.39	2.00	−4.49 (−9.54 to 0.56)	0.08		
PVR										
≥40 ml	139	−4.90	0.58	136	−2.68	0.54	−2.22 (−3.59 to −0.85) *	0.002	0.97	0.87
<40 ml	44	−4.98	0.38	54	−2.63	0.37	−2.35 (−3.15 to −1.55) *	<0.001		

BMI = body mass index; CI = confidence interval; CPSI = Chronic Prostatitis Symptom Index; IIEF = International Index of Erectile Function; LS = least squares; LSTD = LS treatment difference (difference in mLS change from baseline between Xialiqi and placebo); mLS = LS mean; PSA = prostate-specific antigen; PVR = postvoid residual volume; SE = standard error.

a Analysis of covariance was performed using variables that included treatment group, hospital, baseline values, and subgroups as variables, and interactions between treatment and subgroups.

* Statistically significant.

### 3.4 Secondary efficacy measures

**Fig. 3:**
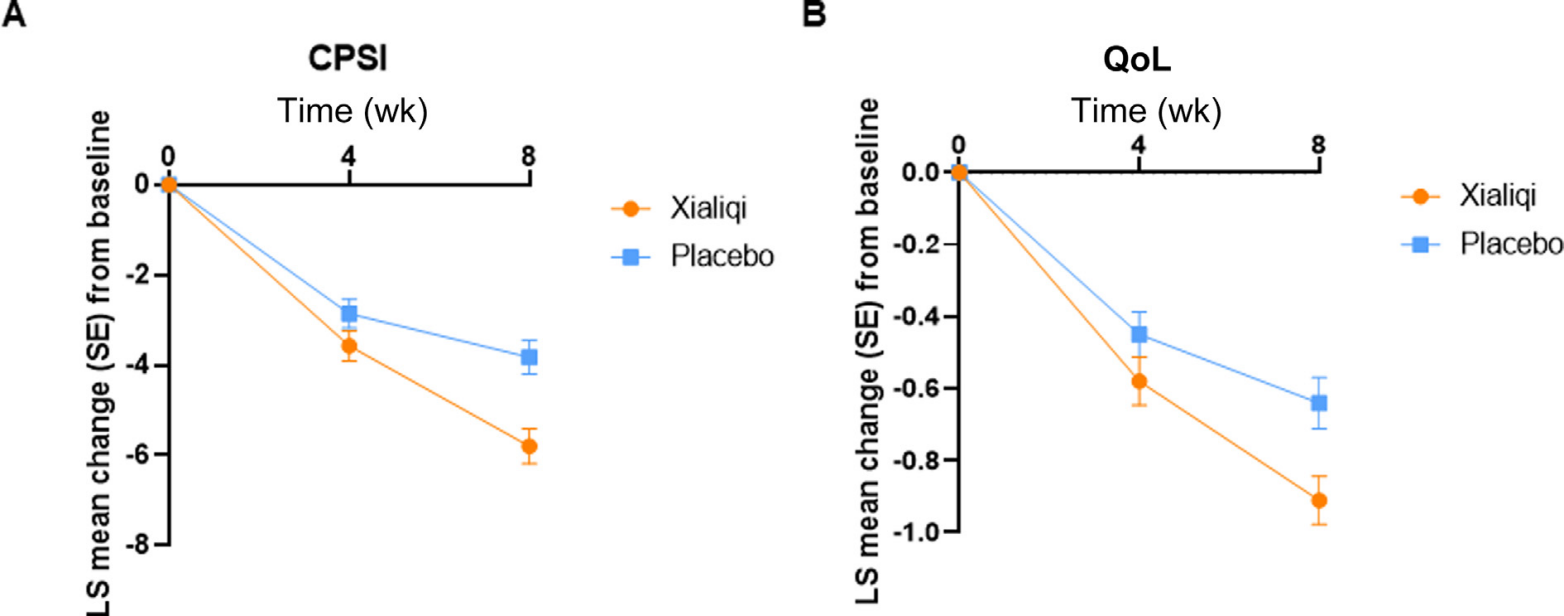
Generalized estimating equation analysis of secondary efficacy variables. Variables in the model included time, treatment, hospital, baseline values, and the interaction between treatment and time. Time was treated as a fixed categorical effect. Least-squares (LS) mean change in (A) Chronic Prostatitis Symptom Index (CPSI) and (B) quality of life (QoL) scores. SE = standard error.

Changes in secondary efficacy measures from baseline were assessed for the two treatment arms via GEE models. At 8 wk, Xialiqi improved prostate volume, Q_max_, Q_ave_, PVR, and IIEF-5 scores in comparison to placebo, although the differences did not reach statistical significance, possibly because of the short therapeutic duration (Supplementary Table 1). Results showed that Xialiqi improved both the Chronic Prostatitis Symptom Index score (*p* = 0.007) and QoL score (*p* = 0.008) to a significantly greater extent than placebo (Fig. 3).

### 3.5 Safety

There were 35 AEs (17.8%) in the Xialiqi arm and 37 AEs (18.7%) in the placebo arm (Supplementary Table 2). Most AEs were mild to moderate in severity. One patient in the Xialiqi arm reported an SAE of intestinal cancer, and two patients in the placebo arm reported SAEs of diffuse pulmonary interstitial fibrosis and dizziness. There were no significant changes in laboratory parameters.

## 4 Discussion

BPH is a common chronic disease among aging men. It is well established that the pathogenesis of BPH is related to epithelial proliferation, inflammation, redox imbalance, and apoptosis [11]. A study in rats indicated that Xialiqi has the potential to prevent the development of testosterone propionate–induced BPH via mechanisms that inhibit inflammation, oxidation, and cell proliferation, and promote apoptosis [12]. Several studies have shown that oxidative stress may also play a role in BPH development in men [13]. Prostate tissue damage and oxidative stress may lead to compensatory cellular proliferation, resulting in hyperplastic growth.

Our results demonstrate that Xialiqi significantly reduced IPSS in a cohort of men with moderate BPH. In addition, Xialiqi improved both CPSI and QoL scores to a greater extent than placebo. Only a few AEs during Xialiqi treatment were reported.

This is the first placebo-controlled multicenter randomized clinical trial to demonstrate the safety and efficacy of Xialiqi, a Chinese herbal product, in patients with BPH. The study revealed a significantly better improvement in total IPSS after 8 wk of treatment with Xialiqi in comparison to placebo.

An improvement in total IPSS was observed in both the Xialiqi and placebo arms. GEE analysis results suggest that Xialiqi reduced IPSS to a greater extent than placebo did in a time-dependent manner when taking treatment, hospital, baseline IPSS, time, and treatment × time interaction into account. Results for irritative IPSS and obstructive IPSS were consistent with those for total IPSS, with improvements in both domains favoring Xialiqi over placebo. In addition, the LS mean change in total IPSS from baseline after 8 wk of Xialiqi therapy (−5.01) was greater than the 12-wk change with finasteride + placebo (−3.8) and similar to the 12-wk change with tadalafil + finasteride (−5.2) in a previous study [5]. The standard deviation for baseline IPSS in our cohort is not particularly large because we only included men with moderate BPH symptoms, and most patients had an IPSS close to the group mean.

The minimal clinically important difference (MCID) in IPSS is generally considered a key indicator of the clinical significance of symptom improvement after treatment. Previous studies have suggested that a minimum improvement of 3 points in IPSS is required for patients to perceive an improvement in LUTS. In some subsequent studies, an improvement of 5 points in IPSS was considered clinically meaningful. The MCID in IPSS may vary according to the specific study design and clinical context. In our study, no patients had particularly severe symptoms, and patients in both arms all received basic LUTS treatment (Supplementary material) and either Xialiqi capsules in the Xialiqi arm, or placebo capsules in the placebo arm. Therefore, the potential improvement in IPSS was smaller than that for patients not receiving basic LUTS treatment. Although the MCID value did not reach 3 points in our study, the improvement in IPSS is statistically significant and meaningful.

In addition, Q_max_ and Q_ave_ increased, whereas prostate volume decreased, after 8 wk of Xialiqi treatment. In a previous study, Q_max_ significantly increased by 3.2 ml/s with 12 wk of alfuzosin 2.5 mg three times daily and by 2.3 ml/s with 12 wk of alfuzosin 10 mg daily [14]. Chapple et al. [15] reported a Q_max_ of 1.6 ml/s after 12 wk of treatment with a daily dose of tamsulosin 0.4 mg. Roehrborn et al. reported that mean Q_max_ increased by 1.2 ml/s from baseline after 12 wk of treatment with tadalafil 5 mg daily [16]. The randomized, placebo-controlled, 4-yr MTOPS trial showed that a combination of doxazosin and finasteride can increase Q_max_ by 3.7 ml/s [5,17]. Consistent with findings in a previous study, Q_max_ significantly increased by 5.12 ml/s after 8 wk of Xialiqi treatment (Supplementary Table 1). This result supports the effectiveness of Xialiqi for treatment of moderate BPH.

There was no statistically significant difference in the LS mean change from baseline between the two arms for prostate volume (*p* = 0.109) or Q_max_ (*p* = 0.053). This lack of difference may be because only men with moderate BPH were included in the trial, and their QoL and erectile function were not severely affected. However, Q_ave_ was significantly greater in the Xialiqi arm than in the placebo arm (2.60 vs 1.90; *p* = 0.020). Moreover, PVR was greater in the Xialiqi arm than in the placebo group (*p* = 0.047). In a previous study, combination tadalafil + finasteride therapy was associated with a mean increase in IIEF score for the erectile function domain of 3.7 at 4 wk and 4.7 at 12 wk [5]. Consistent with findings in the previous study, the LS mean change in IIEF-5 score from baseline to 8 wk was 0.40 in the Xialiqi arm and −0.28 in the placebo arm (*p* = 0.006). Taken together, our results suggest that Xialiqi can improve both LUTS and erectile function.

According to the safety results, the SAE incidence very low and there was no significant difference in AE incidence rate between the Xialiqi and placebo groups (*p* = 0.78). One side effect of Xialiqi is that patients may experience stomach discomfort after taking the capsules. However, Xialiqi may avoid α-blocker and 5-ARI side effects such as impotence, a decrease in libido, and abnormal ejaculation observed in previous studies.

Recent research has shown that traditional Chinese medicines can be used to treat BPH. Feng et al. [18] identified an integrated strategy whereby Yaoshen Gao may regulate the AKT/PI3K pathway and thereby modulate cellular proliferation, fibrosis, inflammation, and angiogenesis. Zhang et al. [19] hypothesized that the traditional Chinese medicine Qianlong Shutong may inhibit the proliferation of cells and the PI3K-AKT signaling pathway. Tang et al. [20] found that Xialiqi was effective in could increasing maximum urinary flow rate effectively. These findings provide insights into the mechanisms underlying the effect of Xialiqi capsules and provide directions for future research.

Our multicenter, randomized, double-blind, placebo-controlled clinical trial with a large BPH cohort assessed the ability of Xialiqi to improve LUTS symptoms. Comparison of Xialiqi to other pharmacotherapeutics or saw palmetto or other antioxidant compounds is a potential avenue for future research, including the ability of Xialiqi to reduce inflammation or redox imbalance. Nevertheless, our study has several limitations. In comparison to previous studies with a treatment duration of 1–4 yr, our trial only lasted for 8 wk, so further investigations over a longer duration are required. The study was designed to observe the short-term efficacy of Xialiqi in improving LUTS in patients with moderate BPH. Another limitation is that only patients with BPH participated in the study, and healthy men without a history of BPH were not included.

## 5 Conclusions

In comparison to placebo, daily therapy with Xialiqi capsules is safe and effective in improving LUTS and prostate enlargement in men with moderate BPH according to total IPSS and irritative and obstructive IPSS subscores. Xialiqi also improved erectile function according to IIEF-5 results, and QoL in terms of Qave and PVR. This is the first randomized controlled trial to demonstrate the safety and effectiveness of Xialiqi in comparison to placebo for the treatment of BPH.

  ***Author contributions***: Nianzeng Xing had full access to all the data in the study and takes responsibility for the integrity of the data and the accuracy of the data analysis.

  *Study concept and design*: Xing.

*Acquisition of data*: All authors.

*Analysis and interpretation of data*: F. Yang, C. Yang, X. Wang.

*Drafting of the manuscript*: F. Yang, C. Yang, X. Wang.

*Critical revision of the manuscript for important intellectual content*: Xing.

*Statistical analysis*: X. Wang.

*Obtaining funding*: Xing.

*Administrative, technical, or material support*: Xing.

*Supervision*: Xing.

*Other*: None.

  ***Financial disclosures:*** Nianzeng Xing certifies that all conflicts of interest, including specific financial interests and relationships and affiliations relevant to the subject matter or materials discussed in the manuscript (eg, employment/affiliation, grants or funding, consultancies, honoraria, stock ownership or options, expert testimony, royalties, or patents filed, received, or pending), are the following: None.

  ***Funding/Support and role of the sponsor*:** The Xialiqi and placebo capsules were provided free of charge by Shijiazhuang Yiling Pharmaceutical Company. The company played a role in the design and conduct of the study.
