# Supplementary material for: Efficacy and Safety of Xialiqi for the Treatment of Benign Prostatic Hyperplasia in a Randomized Trial
Source: Eur Urol Open Sci. 2025 Oct 25;82:73–80. doi: 10.1016/j.euros.2025.10.006 (PMC12596610; doi:10.1016/j.euros.2025.10.006)
Supplement: Supplementary Data 1 [file mmc1.docx]

**Supplementary materials**

Efficacy and Safety of Xialiqi in the Treatment of Benign Prostatic Hyperplasia: A Randomized Trial

Yang Feiya, 1*, Yang Chao, 1,4*, Wang Xin, 2,3*, Chen Dong, 1, Wang Zhong, 5, Wang Jianwen, 6, Liang Chaozhao, 7, Hua Lin, 8, Ping Hao, 9, Lu Jianxin, 10, Wang Zhiping, 11, Li Wei, 12, and Xing Nianzeng, 1**

1. Department of Urology, National Cancer Center/National Clinical Research Center for Cancer/Cancer Hospital Chinese Academy of Medical Sciences and Peking Union Medical College, Beijing, China

2. Department of Clinical Trials Center, National Cancer Center/National Clinical Research Center for Cancer/Cancer Hospital, Chinese Academy of Medical Sciences and Peking Union Medical College, Beijing, China

3. Department of Clinical Trials Center, Shanxi Province Cancer Hospital/Shanxi Hospital Affiliated to Cancer Hospital, Chinese Academy of Medical Sciences/Cancer Hospital Affiliated to Shanxi Medical University, Taiyuan, Shanxi, China

4. Department of Urology, RenJi Hospital, School of Medicine, Shanghai Jiao Tong University, Shanghai, China.

5. Shanghai Ninth People’s Hospital, Shanghai JiaoTong University School of Medicine, China

6. Beijing Chao-Yang Hospital, Capital medical university, China

7. The First Affiliated Hospital of Anhui Medical University, China

8. School of Biomedical Engineering, Capital Medical University, Beijing, China

9. Beijing Tongren Hospital Capital Medical University, China

10. Guang’anmen Hospital China Academy of Chinese Medical Sciences, China

11. The Second Hospital of Lanzhou University, China

12. The Second Hospital of Hebei Medical University, China

* Yang Feiya, Yang Chao, Wang Xin contributed equally to this work.

**Correspondence:

Xing Nianzeng, M.D., Ph.D.

Department of Urology

National Cancer Center/National Clinical Research Center for Cancer/Cancer Hospital, Chinese Academy of Medical Sciences and Peking Union Medical College

No.17, Panjiayuan South Li, Chaoyang, 100021, Beijing, China. Tel: 010-87787170. E-mail address: xingnianzeng@126.com.

**Sample size calculation**

According to the study results of the paper "Efficacy and Safety Evaluation of Tamsulosin in the Treatment of Prostate Hyperplasia" published in the *Journal of Clinical Urology*, volume 26, issue 1, 2011, it was found that the IPSS score of patients treated with basic drugs was 12.60 ± 4.10 ,while in the pre-test, the IPSS score of patients treated with Xialiqi capsule after medication was 11.30 ± 4.10, with α = 0.05 selected, and the test power = 80%. The two-sided test formula designed in parallel with the measurement data of the two groups was adopted according to the 1:1 ratio between the basic medication group and the basic medication + Xialiqi capsule group:


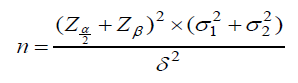


Wherein, σ_1_ and σ_2_ are the standard deviations of the two groups, and δ is the difference in means of the two groups.z$\frac{\alpha}{2}$ and z_β_ represent the quantiles of the standard normal distribution, respectively, with z$\frac{0.05}{2}$ = 1.96, z_0.2_ = 0.84. As calculated by using PASS 13.0 software, the sample size of the basic medication group and the basic medication + Xialiqi capsule group was **156** cases, respectively. Considering that there may be 20% dropouts, loss of follow-up and other reasons, there were **196** patients in the actual basic medication group and 196 patients in the basic medication + Xialiqi capsule group, a total of 392 patients were enrolled.

2

The study is planned to be completed in 10 - 20 sites across the country. Each site will complete 20 - 40 cases.

**Underlying rationnale**

Xialiqi capsule is a kind of listed proprietary Chinese medicine developed and produced by Shijiazhuang Yiling Pharmaceutical Co., LTD. With the main ingredients including Astragalus, glossy privet fruit, talc, selfheal, lychee seed, amber, cinnamon and amur corktree bark, it has the effect of strengthening spleen and kidney, removing water and dispersing knot. It is clinically used in the treatment of mild and moderate benign prostatic hyperplasia, spleen and kidney qi deficiency and phlegm stasis syndrome, which has the symptoms of weak urination, dribbling of urine, frequent urination at night, lower abdomen distension, soreness and weakness of waist and knees, fatigue and lack of strength, etc. Animal studies showed that Xialiqi capsule had good diuretic, anti-proliferation, anti-inflammatory and analgesic effects on animal models of prostatic hyperplasia, chronic bacterial prostatitis and chronic nonbacterial prostatitis ^[11-14]^. Shang Xuejun et al. ^[15-16]^ studied the effects of Xialiqi capsule on the expression levels of proliferating cell nuclear antigen (PCNA), caspase-3, IL-8, TNF-α, DHT, SOD and MDA in model rats with benign prostatic hyperplasia (BPH). It was confirmed that Xialiqi capsule could significantly reduce the wet weight and prostate index of BPH rats, and increase the expression of caspase-3 and SOD in prostate tissue by decreasing the expression levels of PCNA, DHT, MDA, IL-8 and TNF-α in prostate tissue, which may be the mechanism of its treatment of BPH.

**Trial protocol**

**A Multi-center, Randomized, Double-blind, Placebo-controlled Clinical Study of**

**Xialiqi in the Treatment of Benign Prostatic Hyperplasia (BPH)**

**Study Protocol**

**Study Protocol Version No.: 2.0**

**Study Protocol Date of Release: February 22, 2019**

**Leading unit: Cancer Hospital, Chinese Academy of Medical Sciences**

**Principle invesigator: Professor Xing Nianzeng**

**Leader Unit and Principle Investigator**

| **Leader Unit** | **Cancer Hospital, Chinese Academy of Medical Sciences** |
| --- | --- |
| **Principle Investigator** | **Xing Nianzeng** Signature: |

**Member of Academic Board**

| **Member** | **Unit** |
| --- | --- |
| Xing Nianzeng | Cancer Hospital, Chinese Academy of Medical Sciences |
| Ji Zhigang | Peking Union Medical College Hospital, Chinese Academy of Medical Sciences |
| Wang Jianwen | Beijing Chaoyang Hospital Affiliated to Capital Medical University |
| Liang Chaochao | The First Affiliated Hospital of Anhui Medical University |
| Wang Zhong | Ninth People's Hospital Affiliated to Shanghai Jiao Tong University School of Medicine |
| Lu Jianxin | Guang 'anmen Hospital, China Academy of Chinese Medical Science |
| Zhang Yong | Beijing Tiantan Hospital Affiliated to Capital Medical University |
| Ping Hao | Beijing Tongren Hospital Affiliated to Capital Medical University |
| Li Wei | The Second Hospital of Hebei Medical University |
| Lv Jiaju | Shandong Provincial Hospital |
| Zhang Yangang | Shanxi Bethune Hospital, Shanxi Academy of Medical Sciences |
| Yang Guosheng | Shanghai East Hospital |
| Zang Yunjiang | Weifang People’s Hospital |
| Guo Fengfu | Linyi People’s Hospital |
| Liang Taisheng | Ruikang Hospital Affliated to Guangxi University of Chinese Medicine |
| Wang Zhiping | The Second Hospital of Lanzhou University |
| Li Hui | Hebei Yiling Hospital |
| Fan Tiwu | Heping Hospital Affiliated to Changzhi Medical College |

**The 3^rd^ Party Statistical Unit**

| **Data Statistic Unit** | **Responsible Person** | **Telephone** | **E-mail** |
| --- | --- | --- | --- |
| Capital Medical University | Hua Lin | 13661367330 | hualin7750@139.com |

**Summary of Study Protocol**

| **Study title** | A Multi-center, Randomized, Double-blind, Placebo-controlled Clinical Study of Xialiqi in the Treatment of Benign Prostatic Hyperplasia (BPH) | |
| --- | --- | --- |
| **Study objective** | To evaluate the efficacy and safety of Xialiqi capsule in the treatment of benign prostatic hyperplasia.  To explore the therapeutic effect of Xialiqi capsule in benign prostatic hyperplasia complicated with histological prostatitis. | |
| **Study design** | Prospective, randomized, double-blind, placebo-controlled, multi-center clinical study | |
| **Inclusion and exclusion criteria** | **Inclusion criteria:**  (1) Be eligible for the diagnosis of benign prostatic hyperplasia (BPH);  (2) The total score of International Prostate Symptom Score (IPSS) ≥ 8 and ≤ 19;  (3) Prostate volume (PV) ≥ 30 ml;  (4) Maximum urine flow rate < 15 ml/s;  (5) Between 50 and 80 years old (including 50 and 80 years old);  (6) Be willing to participate in clinical trials and sign the informed consent.  **Exclusion criteria:**  (1) Residual urine volume > 150 ml;  (2) Serum PSA > 4 ng/mL;  (3) Severe hepatic and renal insufficiency [ALT ≥ 5 ULN, Cr > 177 μmol/L (2 mg/dl) or eGFR ＜< 45 ml/min/1.73m^2^];  (4) Severe chronic obstructive pulmonary disease (COPD) or respiratory failure;  (5) Serious infection;  (6) Severe weakness, such as cachexia;  (7) Neuropsychiatric disorders;  (8) Malignancies;  (9) Allergic to the ingredients of this study drug;  (10) Patients who are participating in other clinical studies;  (11) Any disease other than benign prostatic hyperplasia (e.g., neurogenic bladder, bladder neck fibrosis, bladder neoplasms, bladder tumors, urinary calculi, urethral stricture, phimosis or penile tumors, acute or chronic prostatitis, prostate cancer, acute or chronic urinary tract infections, acute or chronic renal failure, etc.) that the investigator identifies as causing urination symptoms or changes in urine flow rate;  (12) Other patients deemed unsuitable for this clinical study by the investigator. | |
| **Efficacy measures** | **Primary measures** | The improvement of 8-week IPSS score (compare the changes of International Prostate Symptom IPSS score at week 8 from the mean value at baseline between the two groups) |
|  | **Secondary measures** | 1. Compare the changes of NIH-CPSI score at week 8 from the score at baseline between the two groups;  2. Compare the changes of maximum urine flow rate (Q_max_) at week 8 from the value at baseline between the two groups;  3. Compare the changes of average urine flow rate (Q_ave_) at week 8 from the value at baseline between the two groups;  4. Compare the changes of prostate volume (test by B-ultrasound, formula: V = π/6 × anteroposterior diameter x left-right diameter x suprainferior diameter of prostate) at week 8 from the value at baseline between the two groups;  5. Compare the changes of residual urine volume (RU, test by B-ultrasound, formula: RU = 0.75 × anteroposterior diameter × left-right diameter x suprainferior diameter of bladder) at week 8 from the value at baseline between the two groups;  6. Compare the changes of QOL quality of life score at week 8 from the score at baseline between the two groups;  7. Compare the changes of sexual function evaluation (International Erectile Function Score IIEF-5) at week 8 from the value at baseline between the two groups. |
| **Subgroup analysis** | 1. Pathological examination of prostate (changes in prostate gland morphology and inflammatory cell infiltration, diagnosis of chronic prostatitis by international standards)  2. Changes of NIH-CPSI score, maximum urine flow rate, average urine flow rate, prostate volume, residual urine volume, QOL quality of life score and sexual function score four (4) weeks after surgery. | |
| **Safety**  **Measures** | Physical examination, laboratory examination and adverse events, etc. | |
| **Sample size** | A total of 392 cases were observed, while 196 cases in the trial group and 196 cases in the control group. | |
| **Dosage regimen** | **Trial group**: conventional therapy + taking Xialiqi capsules orally, 3 capsules each time, 3 times a day.  **Control group**: conventional therapy + taking Xialiqi capsule placebo orally, 3 capsules each time, 3 times a day. | |
| **Course of treatment** | 8 weeks | |
| **Number of investigation sites** | 10 - 20 sites | |
| **Expected progress** | December 2021 | |

**Main Body of Study Protocol**

**1. Study Title**

A Multi-center, Randomized, Double-blind, Placebo-controlled Clinical Study of Xialiqi in the Treatment of Benign Prostatic Hyperplasia (BPH)

**2. Study Objectives**

To evaluate the efficacy and safety of Xialiqi capsule in the treatment of benign prostatic hyperplasia. To explore the therapeutic effect of Xialiqi capsule in benign prostatic hyperplasia complicated with histological prostatitis.

**3. Study Background**

The benign prostatic hyperplasia (BPH) is one of the most common benign lesions that cause urinary dysfunction in middle-aged and elderly men, and it is also one of the most common diseases in urology. Its main clinical manifestations include histological hyperplasia of prostatic interstitial and glandular components, anatomical enlargement of prostate volume, urodynamic obstruction of bladder outlet, and the following urinary tract symptoms. Although BPH is a slowly progressing benign disease, as the disease progresses, the lower urinary tract symptoms of patients are gradually aggravated, and acute urinary retention and even renal function impairment can occur in severe cases, which seriously affects the health and quality of life of middle-aged and elderly men.

BPH patients often have some degree of prostatitis, with or without the clinical symptoms of prostatitis, and its histological manifestation is the infiltration or aggregation of inflammatory cells in the prostate tissue. Foreign studies have found that 95% of specimens from transurethral resection of prostate (TURP) of benign prostatic hyperplasia and 100% of total resection specimens have lymphocytes infiltration [^[1]^](#cakao1). Due to the different diagnostic criteria at home and abroad, the detection rate of BPH combined with prostatitis is very different. In the same group of patients, the detection rate of prostatitis using domestic standard was 39.2%, while the detection rate using foreign standard is as high as 89.1% [^[2]^](#cakao2). The author believes that the domestic scholars should refer to the international histological classification of prostatitis and take the inflammatory cell infiltration or aggregation in the prostate tissue as the standard of diagnosis.

For patients with mild to moderate BPH and whose quality of life has not been significantly affected, drug therapy is still the main means of management, with its short-term goal to alleviate the lower urinary tract symptoms of patients, and the long-term goal to delay the clinical progression of the disease and prevent the occurrence of concomitant illnesses and complications. And reducing the side effects of drug therapy while maintaining a higher quality of life for patients is the overall goal of BPH drug therapy. α-blockers, 5-α reductase inhibitors, and a combination of the two are still the most commonly used drugs for BPH[^[3]^](#cakao3), but they are only effective in treating mild to moderate symptoms. Drugs for the treatment of prostate hyperplasia mainly target the enlargement of prostate volume or the increase of smooth muscle tone in both static and dynamic directions, and the drugs targeting the static components of BPH work by inhibiting the proliferation of androgens, which include finasteride and dutasteride, accounting for about 23% of the global prostate drugs [^[4]^](#cakao4). When clinically treated with medications related to 5α reductase inhibitors, it usually works slowly, takes up to 6 months to effectively relieve symptoms, and is associated with sexual side effects, including impotence, decreased libido, and abnormal ejaculation [^[5-7]^](#cakao5). α-adrenergic receptor antagonists are currently the most effective and rapid therapies targeting dynamic components in the treatment of BPH^[8]^, which mainly include Tamsulosin and Tamlosin, accounting for about 65% of the global prostate drugs ^[4]^. These drugs work by reducing the tone of stroma smoothing muscle, thereby reducing urinary tract obstruction and lower urinary tract symptoms ^[9]^. α1-adrenergic receptor antagonists mainly produce side effect of vasodilation, and selective blocking of α1-adrenergic receptor subtypes is also associated with some abnormal ejaculation effects ^[10]^. In addition, many traditional Chinese medicines have a good effect in the treatment of BPH.

Xialiqi capsule is a kind of listed proprietary Chinese medicine developed and produced by Shijiazhuang Yiling Pharmaceutical Co., LTD. With the main ingredients including Astragalus, glossy privet fruit, talc, selfheal, lychee seed, amber, cinnamon and amur corktree bark, it has the effect of strengthening spleen and kidney, removing water and dispersing knot. It is clinically used in the treatment of mild and moderate benign prostatic hyperplasia, spleen and kidney qi deficiency and phlegm stasis syndrome, which has the symptoms of weak urination, dribbling of urine, frequent urination at night, lower abdomen distension, soreness and weakness of waist and knees, fatigue and lack of strength, etc. Animal studies showed that Xialiqi capsule had good diuretic, anti-proliferation, anti-inflammatory and analgesic effects on animal models of prostatic hyperplasia, chronic bacterial prostatitis and chronic nonbacterial prostatitis ^[11-14]^. Shang Xuejun et al. ^[15-16]^ studied the effects of Xialiqi capsule on the expression levels of proliferating cell nuclear antigen (PCNA), caspase-3, IL-8, TNF-α, DHT, SOD and MDA in model rats with benign prostatic hyperplasia (BPH). It was confirmed that Xialiqi capsule could significantly reduce the wet weight and prostate index of BPH rats, and increase the expression of caspase-3 and SOD in prostate tissue by decreasing the expression levels of PCNA, DHT, MDA, IL-8 and TNF-α in prostate tissue, which may be the mechanism of its treatment of BPH.

Therefore, this study was designed to further confirm the efficacy and safety of Xialiqi capsule in patients with benign prostatic hyperplasia.

**4. Study Design**

This study intends to observe the therapeutic effect of Xialiqi capsule on patients with benign prostatic hyperplasia through a multi-center, randomized, double-blinded, placebo-controlled clinical trial, evaluate its effectiveness and safety, and conduct an exploratory analysis on the therapeutic effect in benign prostatic hyperplasia complicated with histological prostatitis through preoperative EPS examination, NIH-CPSI score and postoperative pathological examination.

**4.1. Random allocation**

The central random method was used in this study. Patients who met the inclusion criteria were registered into the central randomization system in the site where they were located, and the relevant information was entered, and the central server automatically performed 1:1 randomization and drug allocation.

Once enrolled, all patients were given either Xaliqi capsule or placebo in addition to basic treatment at the rate of 3 capsules /tid daily for 8 weeks.

**4.2. Sample size evaluation and investigation site allocation**

According to the study results of the paper "Efficacy and Safety Evaluation of Tamsulosin in the Treatment of Prostate Hyperplasia" published in the *Journal of Clinical Urology*, volume 26, issue 1, 2011, it was found that the IPSS score of patients treated with basic drugs was 12.60 ± 4.10 ,while in the pre-test, the IPSS score of patients treated with Xialiqi capsule after medication was 11.30 ± 4.10, with α = 0.05 selected, and the test power = 80%. The two-sided test formula designed in parallel with the measurement data of the two groups was adopted according to the 1:1 ratio between the basic medication group and the basic medication + Xialiqi capsule group:


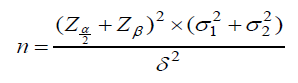


Wherein, σ_1_ and σ_2_ are the standard deviations of the two groups, and δ is the difference in means of the two groups.z$\frac{\alpha}{2}$ and z_β_ represent the quantiles of the standard normal distribution, respectively, with z$\frac{0.05}{2}$ = 1.96, z_0.2_ = 0.84. As calculated by using PASS 13.0 software, the sample size of the basic medication group and the basic medication + Xialiqi capsule group was **156** cases, respectively. Considering that there may be 20% dropouts, loss of follow-up and other reasons, there were **196** patients in the actual basic medication group and 196 patients in the basic medication + Xialiqi capsule group, a total of 392 patients were enrolled.

2

The study is planned to be completed in 10 - 20 sites across the country. Each site will complete 20 - 40 cases.

**5. Study Population**

To enroll, patients must meet all of the inclusion criteria listed below and must exclude any of the exclusion criteria.

**5.1. Inclusion and exclusion criteria**

**5.1.1. Inclusion criteria**

1. Be eligible for the diagnosis of benign prostatic hyperplasia (BPH);

2. The total score of International Prostate Symptom Score (IPSS) ≥ 8 and ≤ 19;

3. Prostate volume (PV) ≥ 30ml (nuclear magnetic resonance method);

4. Maximum urine flow rate ＜ 15 ml/s;

5. Between 50 and 80 years old (including 50 and 80 years old);

6. Be willing to participate in clinical trials and sign the informed consent.

**5.1.2. Exclusion criteria**

1. Residual urine volume > 150 ml;

2. Serum PSA > 4 ng/mL;

3. Severe hepatic and renal insufficiency [ALT ≥ 5 ULN (Upper Limit of Normal), Cr >177 μmol/L (2 mg/dl) or eGFR < 45 ml/min/1.73m^2^];

4. Severe chronic obstructive pulmonary disease (COPD) or respiratory failure;

5. Serious infection;

6. Severe weakness, such as cachexia;

7. Neuropsychiatric disorders;

8. Malignancies;

9. Allergic to the ingredients of this study drug;

10. Patients who are participating in other clinical studies;

11. Any disease other than benign prostatic hyperplasia (e.g., neurogenic bladder, bladder neck fibrosis, bladder neoplasms, bladder tumors, urinary calculi, urethral stricture, phimosis or penile tumors, acute or chronic prostatitis, prostate cancer, acute or chronic urinary tract infections, acute or chronic renal failure, etc.) that the investigator identifies as causing urination symptoms or changes in urine flow rate;

12. Other patients deemed unsuitable for this clinical study by the investigator.

**5.2. Dropout (withdrawal) criteria**

All subjects who filled in the informed consent form and were screened for admission into the trial were called drop-out cases, regardless of when and why they withdrew, as long as they did not complete the protocol.

Patients have the right to withdraw from the study at any time and for any reason. The investigator also has the right to withdraw patients from the study if they are not applicable to continue the study because of illness, adverse events, violation of the study protocol, poor compliance, or other reasons. Unnecessary patient withdrawal should be avoided as much as possible and remedial measures should be actively taken. If the patient has decided to withdraw, the last test (examination) should be completed as far as possible for the analysis of efficacy and safety; The investigator should contact the patient or their responsible relatives by telephone or personal interview to confirm the reason for withdrawal as much as possible and to recover the remaining drugs. When advising patients to withdraw from the study, the investigator should explain the reasons for withdrawal to the patients or their responsible relatives, and maintain follow-up of the endpoint events in the patients who withdrew, complete the final assessment and case report. If the reason for withdrawal is an adverse event, the major events should be recorded on the CRF table and the follow-up should be up to 8 weeks if possible.

Common causes of dropout: adverse events, lack of efficacy, violation of trial protocol (including poor compliance), loss of follow-up (including patient withdrawal), discontinuation, and others.

**5.3. Criteria for trial termination**

(1) the occurrence of allergic reactions clearly related to the investigational drug;

(2) The occurrence of adverse symptoms or signs and abnormal test results clearly related to the study drug, and the conditions in which the investigator determined that the study should be terminated;

(3) The study was terminated at the patient's request.

**5.4. Criteria for complete discontinuation of the study**

(1) During the study, the entire trial was stopped in multiple sites for the following reasons:

1) The investigator found serious safety issues;

2) The efficacy was too poor to continue the trial;

3) The protocol has a major failure;

4) Irresistible reasons of the study’s sponsor;

5) If the competent administrative department revokes the study, it may stop all the trials midway.

(2) The complete discontinuation of the study can be temporary or permanent. When discontinuing the study, all the study records shall be retained for future reference.

**6. Therapy**

**6.1. Basic therapy**

According to the *Guidelines for Diagnosis and Treatment of Benign Prostatic Hyperplasia* and hospital medication experience, patients can be given life-style change / observation and waiting, drug therapy, minimally invasive treatment, and surgical treatment.

(1) Recommendations for lifestyle changes include limiting fluid intake before bedtime and avoiding caffeinated beverages, alcohol and spicy foods, etc.

(2) Drug treatment can include α-blockers, 5α reductase inhibitors (5ARIs), α-blockers combined with 5ARIs, long-acting phosphodiesterase inhibitors, etc.

(3) Minimally invasive treatment and surgery can be performed after 8 weeks of oral administration of the study drug without affecting the patient's condition. Patients who were expected to undergo minimally invasive treatment and surgery within 8 weeks were excluded from the study.

**6.2. Investigational drug**

(1) Test drug: Xialiqi capsule

1) Ingredients: Astragalus, glossy privet fruit, talc, selfheal, lychee seed, amber, cinnamon and amur corktree bark

2) Description: This product is a hard capsule, with the content of brown to sepia particles; slightly fragrant and slightly bitter.

3) Strength: 0.45 g per capsule

4) Manufacturer: Shijiazhuang Yiling Pharmaceutical Co., Ltd.

(2) Placebo: it is completely consistent with the color, strength, packaging, label, contents and shape of Xaliqi capsules, but does not contain active pharmaceutical ingredients.

All the above-mentioned test drugs and placebos are provided free of charge by Shijiazhuang Yiling Pharmaceutical Co., Ltd., and a qualified drug test report is issued.

**6.2.1. Drug product, packaging and label**

(1) The Xialiqi capsule will be delivered in 0.45g capsule form with placebo appearance identical to that of the capsule.

(2) Drug packaging

1) Small package: The appearance is as follows, printed with the words "clinical study drug", each small box contains 36 capsules packed in aluminium-plastic panel.

2) Large package: white board packaging, each package contains 16 small packages, for 8-week use.

(3) Label

| **Clinical study drug Xialiqi in the treatment of benign prostatic hyperplasia (BPH)** |
| --- |
| (Use for clinical study only) |
| **Drug packaging No.: XXXXX**  [Product batch number] xxxx [Expiration date] xxxx |
| [National Medical Products Administration Approval No.] Z20123085 |
| [Packaging] 36 capsules each small box, and 16 small boxes inside |
| [Dosage and administration] 3 times daily, 3 capsules each time |
| [Storage] Seal and store in a dry place, out of reach of children |
| **Please be sure to follow the doctor's orders and visit the hospital the specified date, thank you for cooperation!** |

**6.2.2. Preservation method**

The test drug should be locked and stored in a safe and controllable indoor area, and pay attention to sealing and moisture-proof. Each investigation site must designate a test drug management personnel for preservation and management.

**6.2.3. Drug dispensing and recycling**

In this study, the random number would be assigned centrally. After the subjects were qualified, the investigator logged in to the central randomization system, entered relevant information of the subjects to apply for a randomization number, and filled the subject's randomization number in the "Randomization Number" column of the study case report form (CRF). The drug dispenser shall check the package number on the outer package of the drug with the package number displayed in the system before dispensing the drug.

When dispensing drugs, investigators should fill in the drug dispensing registration form promptly and accurately. The quantity and package number of drugs dispensed shall be recorded promptly and accurately. At the end of the study, the remaining drugs should be returned to the drug supplier and the study drug recovery form should be filled in. The undispensed drugs of each investigational unit must be sealed at the time of return. After the end of the study, the remaining drugs shall be recovered by the drug supplier for unified treatment.

**6.2.4. Medication compliance**

During each follow-up visit, observe whether the doctor recorded in detail whether the patient went to the designated place (outpatient clinic) on time for examination and treatment, so as to judge the subject's compliance. And record it on the formal medical record timely. Medication compliance = actual dosage ÷ total dosage required by protocol × 100%.

**6.2.5. Drug delivery**

The central randomization method was used in this study. Appropriate drugs were first delivered to each investigation site according to the expected progress of the study, and then timely delivered according to the actual progress during the study, and then distributed to the subjects by the test drug management personnel of the investigation site. Each drug distribution process should be recorded accordingly. In order to ensure the timely supply of drugs, the central random system pre-sets the amount of drug warning, once the stock is insufficient, the drug supplier can timely distribute drugs according to the number prompted by the system. The system will not release the drug to the applicant until it is confirmed that the drugs have arrived at the investigation site. Follow the same procedures when dispensing drugs between investigation sites. After the end of the study, the test drug administrator shall be responsible for returning the remaining drugs to the drug supplier for destruction according to the procedures. The test drugs should be locked in the counter and kept sealed at room temperature.

**6.3. Treatment regime**

**6.3.1. Medication process**

(1) Visit 1 (Day -5 - Day 0)

- After the first medical contact with the patient, the investigator completed relevant examinations in a timely manner and gave full informed consent. For patients who met the inclusion criteria and did not meet the exclusion criteria, the investigator voluntarily participated and signed the informed consent letter by himself or his immediate family members, then applied for the random number and drug package number in the central random system and dispensed drugs.
- Collect general information: name, date of birth, height, weight, waistline, etc.;
- Laboratory examinations (including: maximum urine flow rate (Q_max_), average urine flow rate (Q_ave_); prostate volume, residual urine volume (RU), urine routine, blood routine, PSA, blood biochemistry, etc. If the patient has been examined, there is no need to repeat the examination);
- Symptom score (including: IPSS score, NIH-CPSI score, QOL quality of life score, IIEF-5 score, etc.);
- Physical examination;
- Record comorbidities and concomitant medication;
- Review inclusion criteria and exclusion criteria;
- Patients receive study drugs;
- Instruct subjects of next follow-up date.

The study drug is recommended to be taken approximately 30 minutes after three meals a day. If the patient does not take the medicine on one day, the dose of the next day shall not exceed the daily dose. The dose modification is not allowed during the study. If a patient experiences an adverse event that is intolerable and, according to the investigator's consideration, is related to the study drug, the patient should discontinue the study drug treatment.

(2) Visit 2 (4 weeks after medication)

- Adverse event record;
- Laboratory examinations (including: maximum urine flow rate (Q_max_), average urine flow rate (Q_ave_); prostate volume, residual urine volume (RU), urine routine, blood routine, blood biochemistry, etc. If the patient has been examined, there is no need to repeat the examination);
- Symptom score (including: IPSS score, NIH-CPSI score, QOL quality of life score, IIEF-5 score, etc.);
- Physical examination;
- Record concomitant medication;
- Evaluate the medication compliance.

(3) Visit 3 (8 weeks after medication)

- Adverse event record;
- Laboratory examinations (including: maximum urine flow rate (Q_max_), average urine flow rate (Q_ave_); prostate volume, residual urine volume (RU), urine routine, blood routine, blood biochemistry, etc. If the patient has been examined, there is no need to repeat the examination);
- Symptom score (including: IPSS score, NIH-CPSI score, QOL quality of life score, IIEF-5 score, etc.);
- Physical examination;
- Record concomitant medication;
- Evaluate the medication compliance;
- Recover the remaining test drugs.

(4) Visit 4 (after TUR-P surgery)

- Pathological examination after electro-prostatectomy.

(5) Visit 5 (4 weeks after TUR-P surgery)

- Adverse event record;
- Laboratory examinations (including: maximum urine flow rate (Q_max_), average urine flow rate (Q_ave_); prostate volume, residual urine volume (RU), urine routine, blood routine, blood biochemistry, etc. If the patient has been examined, there is no need to repeat the examination);
- Symptom score (including: IPSS score, NIH-CPSI score, QOL quality of life score, IIEF-5 score, etc.);
- Physical examination;
- Record concomitant medication.

**6.3.2. Description of basic therapy**

The investigators refer to the basic treatment regime recommended by the guidelines according to the patient’s condition. All relevant basic therapy drugs should be recorded in detail in the case report form.

**6.3.3. Concomitant medication**

(1) After entering the randomization stage, traditional Chinese medicine or proprietary Chinese medicine other than the study drug with similar functions to the study drug shall not be used during the whole treatment period.

(2) If patients were using Xaliqi capsule or traditional Chinese medicine with similar functions to Xaliqi capsule before enrollment, they should stop taking the drug for 2 weeks before enrollment.

(3) Any drug co-administered after enrollment must be recorded on the case report form with the drug name, reason for use, method and dose taken, and the duration of treatment.

**6.3.4. Course of treatment**

8 weeks

**6.4. Compliance evaluation**

The compliance of the subjects was evaluated through the complete record of drug distribution and recovery. If tthe actual drug dosage was within the range of 80% - 120% of the applied drug dosage, the medication compliance could be determined to meet the requirements of the protocol.

**6.5. Adverse drug reaction**

Xialiqi capsule has less adverse reactions, individual patients after taking the drug will experience stomach upset symptoms, which can be mitigated when taking after meals instead.

**7. Clinical efficacy measures and endpoint events**

**7.1. Clinical efficacy measures**

**7.1.1. Primary efficacy measures**

The improvement of 8-week IPSS score (compare the changes of International Prostate Symptom IPSS score at week 8 from the mean value at baseline between the two groups).

**7.1.2. Secondary efficacy measures**

1. Compare the changes of NIH-CPSI score at week 8 from the score at baseline between the two groups;

2. Compare the changes of maximum urine flow rate (Q_max_) at week 8 from the value at baseline between the two groups;

3. Compare the changes of average urine flow rate (Q_ave_) at week 8 from the value at baseline between the two groups;

4. Compare the changes of prostate volume (test by B-ultrasound, formula: V = π/6 × anteroposterior diameter × left-right diameter × suprainferior diameter of prostate) at week 8 from the value at baseline between the two groups;

5. Compare the changes of residual urine volume (RU, test by B-ultrasound, formula: RU = 0.75 × anteroposterior diameter × left-right diameter × suprainferior diameter of bladder) at week 8 from the value at baseline between the two groups;

6. Compare the changes of QOL quality of life score at week 8 from the score at baseline between the two groups;

7. Compare the changes of sexual function evaluation (International Erectile Function Score IIEF-5) at week 8 from the value at baseline between the two groups.

8. Perform subgroup analysis on prostate cases and postoperative symptom improvement.

**7.2. Safety measures include**

- Adverse event evaluation
- Laboratory measures
- Clinical physical examination

**8. Observation of adverse event**

**8.1. Definition of adverse event**

- Adverse events (AEs): Any adverse medical event that occurred between the time the subject signed the informed consent and was enrolled in the study and the last follow-up, regardless of whether there was a causal relationship with the study drug, was considered an adverse event.
- Serious adverse events (SAEs): Events occurred during clinical studies that require hospitalization or prolonged hospitalization, cause permanent or significant disability, affect the ability to work, threaten life or death, or cause congenital malformations or birth defects were considered as serious adverse events.
- Significant adverse events: In addition to serious adverse events, any occurrence of adverse events and hematological and/or other laboratory abnormalities that result in targeted medical interventions such as drug discontinuation, dose reduction, and symptomatic treatment.

**8.2. Judgment criteria for intensity of adverse events**

All clinical adverse events (except specific adverse events) that occur in this clinical study will be recorded on the Adverse Events page of CRF. And the intensity of the adverse events will be graded. To unify the standard, the intensity of the event is graded as follows:

(1) Mild: Perceptible discomfort but with no influence on daily activities;

(2) Moderate: A strong sense of discomfort that interferes with or reduces daily activities;

(3) Severe: Unable to work or carry out daily activities.

Pay attention to distinguish the severity and intensity of adverse events. Severe is used to describe the intensity and is not necessarily a serious adverse event (SAE). For example, a headache may be severe in intensity but cannot be classified as a serious adverse event unless it meets SAE standards.

**8.3. Criteria for Judging the Relationship Between Adverse Events and Study Drugs**

The causal analysis of the relationship between all adverse events and the study drug was judged according to five grades: definitely relevant, very likely relevant, possibly relevant, possibly irrelevant, and definitely irrelevant, and the first three were classified as adverse reactions of the drug. The considerations of causal analysis include the following five aspects:

(1) Whether there is a reasonable relationship between the time of initiation of medication and the time of occurrence of an adverse drug reaction (ADR) (appear when taking drugs).

(2) Whether the suspected ADR is consistent with the known ADR of the drug (compliance with the literature).

(3) Whether the suspected ADR can be explained by the concomitant medication, previous drugs, the patient's clinical condition, or the effects of other therapies (other explanations).

(4) Whether the suspected ADR disappears or mitigates after discontinuation or reduction (withdrawal reaction).

(5) Whether the suspected ADR reappears after re-exposure to the same drug (reappear when taking again).

Investigators should evaluate possible associations between adverse events and study drug and concomitant drug, as shown in the table below

| Considerations | Appear when taking drugs | Compliance with the literature | Other explanations | Disappear after withdrawal | Reappear when taking again |
| --- | --- | --- | --- | --- | --- |
| Definitely relevant | ＋ | ＋ | － | ＋ | ＋ |
| Very likely relevant | ＋ | ＋ | － | ＋ | ？ |
| Possibly relevant | ＋ | ＋ | ± | ± | ？ |
| Possibly irrelevant | ＋ | － | ± | ± | ？ |
| Definitely irrelevant | － | － | ＋ | － | － |

**8.4. Judgment of serious adverse events**

**8.4.1. Definition of general serious adverse event**

A serious adverse event is any clinical event that suggests a significant hazard, contraindication, side effect, or caution. Adverse events are classified as serious adverse events if they meet one or more of the following criteria:

- Death
- Life threatening, indicating that the patient in question was at immediate risk of death at the time of the event; It does not include events that, if more severe, would likely result in death
- Causing persistant or significant labor incapacity or disability
- Congenital malformations or defects
- Causing hospitalization or an extended stay

Some medical events that have not yet resulted in death, life threatening, or hospitalization are also considered SAE when appropriate medical judgment indicates that they may cause harm to the patient or subject or require drug or surgical treatment to prevent such an occurrence.

**8.5. Abnormal laboratory results**

The investigator should determine whether the abnormal laboratory results are clinically significant and provide possible explanations. Abnormal laboratory results resulting from reported adverse events should also be recorded as adverse events in the Adverse Event table. Clinically significant laboratory abnormalities that meet one or more of the following criteria should be recorded on the Adverse Events page of the CRF as an independent diagnosis (excluding laboratory abnormalities due to reported adverse events):

- Abnormal results with clinical symptoms
- Abnormal results resulting in changes in study medication
- Abnormal results that need to change the concomitant medication and/or other treatment measures

**9. Blinding and randomization**

**9.1. Random allocation method of subjects**

The central random allocation method was used in this study. Patients who met the inclusion criteria after emergency examination were registered into the central randomization system in the site where they were located, and the relevant information was entered, and the central server automatically performed 1:1 randomization and drug allocation.

**9.2. Blinding**

The blinding method in this study was double-blinded, that is, both patients and clinical investigators were blinded.

**9.3. Urgent unblinding**

In the event of an adverse event, emergency unblinding should be performed only in exceptional cases where it is necessary to understand the use of the study drug to treat the patient. Once the decision is made to unblind, the investigator must record the date, time, and reason for breaking of blindness.

The investigator must log in to RTSM to fill in the unblinding application, which is reviewed by the principle investigator and then performed breaking of blindness by the blindness breaking personnel. Once the blindness is broken, the case is discontinued from study and treated as dropout.

**9.4. Unblinding rule**

In this study, the double unblinding method was used. After the blinding state check, the data is locked, and the principle investigator, medical statistician and data administrator perform the first unblinding, and the corresponding groups of each random number are marked with the code A and B, so as to carry out statistical analysis on all the data. When the statistical analysis was done and the statistical report was completed, the second unblinding was performed and the exact groups of group A and Group B were announced.

**10. Data Management**

In this study, Epidata 3.1 (or higher) software was used to collect the study data. The data management ensures the authenticity, integrity and accuracy of clinical trial data, data management process should comply with the requirements of *Good Clinical Practice*, *Technical Guidance for Clinical Trial Data Management* and other regulations, to ensure the traceability of clinical trial data. The main processes of data management are listed below.

**10.1. Design of database**

The data administrator designs the database using Epidata 3.1 software (higher version or EDC) according to CRF, and releases it after testing.

**10.2. Data entry**

CRC is responsible for entering the data in the CRF into the database, and the data is entered twice by two CRCs, respectively. The data administrator compares the two databases and generates a list of data inconsistencies. CRCs modify the respective databases according to the list and compare with the CRF again. Repeat the previous steps until the two databases are identical.

**10.3. Data query management**

The data administrator writes the data verification SAS program according to the data verification plan (DVP) to verify the data and generate the data query list. After manual verification, the data query form is generated, and the CRA submits the query form to the investigator for question answering. After the question answering, the query form is returned by the CRA to the data administrator who will revise the database accordingly.

**10.4. Medical coding**

Adverse events are coded with MedDRA 21.0 (or higher).

**10.5. Data review**

After the database cleaning is completed, the data administrator writes the *Data Verification Report* for the data verification meeting. The key records of the review report include: number of enrolled cases, case dropouts, cases excluded, deviation from or violation of the protocol, compliance data, concomitant medication, adverse events, and the data related to evaluation indicators.

At the data review meeting, the division of the statistical population was discussed and determined according to the content of the review report.

**10.6. Database locking and data export**

Complete the database locking list and complete the database locking according to the database locking procedure. Problems found after data locking can be corrected in the statistical analysis program after confirmation. After the data is locked, if there is conclusive evidence that it is necessary to unlock, the investigator and relevant personnel need to sign the unlocking document.

After the database is locked, the data administrator exports the data file in SAS format and sends it to statisticians for statistical analysis.

**10.7. Data quality audit**

After data cleaning is completed, QA will check the data quality, perform 100% inspection on the critical data (important data determined by the study protocol), and for the non-critical data, select a certain range of data for spot checks, and form a final data quality audit report. The following is a flow chart of data management.

| Data transfer and protocol training |
| --- |
| ↓ |
| Database building and inspection |
| ↓ |
| Database test and release |
| ↓ |
| Data entry, audit and quality mining |
| ↓ |
| Database cleaning |
| ↓ |
| Data review report and review meeting |
| ↓ |
| Data audit list |
| ↓ |
| Correct and lock database |
| ↓ |
| Data management report |

**12. Statistical analysis**

**12.1. Analysis dataset**

- Full analysis set (FAS): Refers to the dataset obtained by removing subjects from all randomized subjects in a minimum and reasonable way. Exclusions usually include: violation of critical inclusion criteria; the subjects not being treated with the study drug; no observational data available after randomization. FAS is the main analysis set.
- Per protocol set (PPS): a subset of the full analysis set, the subjects in this analysis set are more compliant with the protocol. Subjects included in PPS generally have the following characteristics: (1) completion of the preset minimum exposure to the study drug, that is, 80% compliance to the drug; (2) The data of main measures in the study can be obtained; (3) There is no major deviation to the study protocol. PP analysis is mainly used for primary efficacy measures.
- Safety dataset (SS): all subjects who received at least one treatment after randomization and were evaluated for safety.

**12.2. Statistical method**

After the study protocol is determined, the statistical professional is responsible for preparing the statistical analysis plan in consultation with the principal investigator. The statistical analysis software is SAS 9.4 (or higher). The sample size is calculated using Software PASS 13.0.

All statistical tests were conducted using a two-sided test, and a P-value less than or equal to 0.05 would be considered statistically significant for the difference tested. (Unless otherwise specified)

The description of quantitative measures will calculate the mean, standard deviation, median, minimum, maximum, lower quartile (Q1), upper quartile (Q3), and classification indicators describe the number and percentage of each type of case.

The comparison of the general conditions between two groups will be analyzed by appropriate methods according to the type of indicators. The group t test or Wilcoxon rank sum test will be used to compare quantitative data between groups, the Chi-square test or exact probability method for classified data, and the Wilcoxon rank sum test or CMH test will be used for rank data.

**12.3. Case enrollment analysis**

- List the number of cases included and completed in total and in each site, identify the three analysis datasets (FAS, PPS, SS).
- List the dropout and exclusion cases of each site and the reasons.

**12.4. Demographic data and baseline analysis**

Descriptive demographic data and other baseline characteristic values:

- For continuous variables, calculate their number of cases, mean, standard deviation, median, minimum, and maximum.
- For counting and ordinal data, calculate the frequency and composition ratio.
- For inferential statistical results (P-values), list them as descriptive results.

**12.5. Efficacy analysis**

Analyze the primary and secondary measures related to efficacy. For continuous variables, calculate their number of cases, mean, standard deviation, median, minimum, and maximum. For counting and ordinal data, calculate the frequency and composition ratio. It is mainly to evaluate the improvement of IPSS scores in the two groups and compare them between the two groups.

**12.6. Safety analysis**

First, according to the requirements of the correlation of adverse reactions, describe the adverse events and adverse reactions of the two groups in the form of a list (including the number of adverse events of various kinds, and the laboratory test indicators of "normal to abnormal" or "abnormality intensification" before and after the study are recorded as adverse events). Perform statistical analysis on the incidence of adverse events between the groups.

**13. Interim Analysis**

No interim analysis was performed in this study

**14. Quality Control**

**14.1. Measures to improve observational consistency**

(1) The personnel participating in the observation and collection of clinical data should have high professional knowledge and skills, and be relatively fixed.

(2) With the training before clinical study, investigators will have a full understanding of the clinical study protocol and the specific connotation of each indicator. The description of symptoms should be objective, do not induce or prompt; and the specified objective indicators should be checked in accordance with the time and method specified in the protocol. The adverse reactions and unexpected side effects should be observed closely, and the observations should be tracked. The medical record form cannot be altered arbitrarily (When it is necessary to modify, it should be modified according to the required standard method).

(3) Data that deviate from or fall outside the acceptable range significantly must be verified and made necessary explanations by the investigator.

(4) Each test item must be indicated with the unit of measurement used.

(5) Each clinical study unit shall organize a research team. In addition to the need for a unit PI to assume the overall responsibility, a special person should be designated as the general coordinator of the project to help the unit PI to coordinate the cooperation between clinical and imaging departments, regularly check the progress of clinical trials, carefully supervise and verify the timely record of data.

(6) When necessary, the unit in charge of the project will organize a clinical coordination teleconferencing or video conference to check the preliminary work in time, analyze the problems found and existing in the clinical trial process, and propose rectification plans and specific measures to ensure the normalized and smooth implementation of the project.

**14.2. Quality control requirements of laboratory**

Each participating hospital laboratory (or department of clinical laboratory) shall, in strict accordance with national regulations and standards, establish standard operating procedures and quality control systems for research and measurement of indicators. When the main indicators may be subject to subjective influence, consistency testing should be carried out. When the test results of the laboratories of all site are greatly different or the normal reference value ranges are different, effective measures should be taken to verify or correct in time to avoid the test deviation.

**14.3. Measures to ensure subject compliance**

(1) Make the subjects understand the significance of the trial, and ask family members to urge them to take medication. A subject manual should be issued to each subject upon discharge, including information on study and treatment, medication and precautions after discharge, and specific time, location and requirements for each follow-up visit.

(2) It is best to establish a Wechat consultation group of patients participating the study or their families in order to communicate and explain the relevant study and clinical problems encountered in the study and follow-up in a timely manner.

(3) For patients with poor efficacy and those who cannot take medication on time, it is especially necessary to understand and analyze the reasons, and strengthen follow-up.

This study requires the relevant responsible unit to dispatch or entrust clinical study supervisors to ensure that the rights and interests of the subjects in the clinical trial are protected, that the trial records and reports are accurate and complete, and that the trial complies with the approved protocols, the *Good Clinical Practices* and relevant regulations.

**15. Ethical matters**

**15.1. Ethics review**

This clinical trial must comply with the *Declaration of Helsinki* and relevant regulations of clinical trial in China. Before the study starts, the study protocol shall be reviewed by the ethics committee of the responsible research unit hospital and the approval document shall be issued before the implementation of the study protocol. The opinion of the Ethics Committee may be: agree, agree with the necessary amendments, disagree, terminate or suspend the approved trial.

**15.2. Subject informed consent**

Before each patient is enrolled in the study, the study physician shall provide him/her (or his/her designated representative) with a complete and comprehensive written description of the purpose, nature, procedure, and possible benefits and risks of the study. Patients should be made aware of their rights to withdraw from the study at any time. Prior to enrollment, each patient must be given a written informed consent so that the subject can give consent after understanding. Patients can be enrolled in the clinical trial only after they voluntarily participate and have their own or immediate family members sign the informed consent. The informed consent should be kept as one of the original data of clinical trials for future reference.

**16. Trial progress**

**17. Data storage**

All participating study unit hospitals shall, in accordance with the requirements of the Good Clinical Practices (GCPs), keep these original data until 5 years after the termination of the clinical trial, including the confirmation of the participation of all subjects (can effectively check different records, such as CRF form and original records of hospital medical records), informed consents of all subjects, CRF form, detailed records of drug distribution and follow-up results.

**18. Clinical Summary**

After the end of statistical analysis, the statistical unit shall make statistics on the trial data, issue a statistical report and seal it.

**References**

1. Blumenfeld W, Tucci S and Narayan P. Incidental lymphocytic prostatitis. Selective involvement with nonmalignant glands. The American journal of surgical pathology. 1992; 16(10):975-981.

2. Zhang Xianghua, Zhang Qian, Li Xuesong, Zhang Zheng, Yang Xinyu and He Qun. The detection rate of benign prostatic hyperplasia combined with histological prostatitis: a comparative study of two different diagnostic criteria. Chinese Journal of Clinicians (Electronic Edition). 2007; 1(7):29-31.

3. Letteria Minutoli , Mariagrazia Rinaldi , Herbert Marini. Apoptotic Pathways Linked to Endocrine System as Potential Therapeutic Targets for Benign Prostatic Hyperplasia[J]. International Journal of Molecular Sciences, 2016,17:1-15.

4. S Ventura,VL Oliver,CW White,etal.Novel drug targets for the pharmacotherapy of benign prostatic hyperplasia (BPH)[J]. British Journal of Pharmacology,2011,163:891-907.

5. Adolfo Casabe,Claus G. Roehrborn,Luigi F. Da Pozzo，etal. Efﬁcacy and Safety of the Coadministration of Tadalaﬁl Once Daily with Finasteride for 6 Months: A Randomized, Double-Blind, Placebo Controlled Study in Men with Lower Urinary Tract Symptoms and Prostatic Enlargement Secondary to Benign Prostatic Hyperplasia[J]. THE JOURNAL OF UROLOGY,2014, 3(191):1-7.

6. Carson C, 3rd, Rittmaster R (2003). The role of dihydrotestosterone in benign prostatic hyperplasia. Urology 61: 2-7.

7. Tarter TH, Vaughan ED, Jr (2006). Inhibitors of 5alpha-reductase in the treatment of benign prostatic hyperplasia. Curr Pharm Des 12: 775-783.

8. Miano R, De Nunzio C, Asimakopoulos AD, Germani S, Tubaro A (2008). Treatment options for benign prostatic hyperplasia in older men. Med Sci Monit 14: 94-102.

9. Lepor H. Alpha blockers for the treatment of benign prostatic hyperplasia. Rev Urol ,2007(9): 181-190.

10. Rokosh DG, Simpson PC (2002). Knockout of the alpha 1A/C-adrenergic receptor subtype: the alpha 1A/C is expressed in resistance arteries and is required to maintain arterial blood pressure. Proc Natl Acad Sci USA 99: 9474– 9479.

11. Hong Xiaohua, Liu Jianxun, Yu Weilin, Li Hongkun and Dong Xiaoxia. Pharmacological study I of Qianlieshu capsule in the treatment of prostatitis——Effect on experimental bacterial prostatitis rat model. Chinese Journal of Experimental Traditional Medical Formulae. 2010; 16(10):122-124.

12. Hong Xiaohua, Liu Jianxun, Yu Weilin, Li Hongkun and Dong Xiaoxia. Pharmacological study II of Qianlieshu capsule in the treatment of prostatitis——Effects on experimental nonbacterial prostatitis, inflammation, pain and diuretic animal models. Chinese Journal of Experimental Traditional Medical Formulae. 2010; 16(12):105-108.

13. Hong Xiaohua, Wang Qin, Li Hongkun and Liu Jianyun. Effect of Qianlieshu capsule on experimental prostatic hyperplasia model. Chinese Journal of Experimental Traditional Medical Formulae. 2008; 14(2):60-62.

14. Hong Xiaohua, Wang Qin, Yu Weilin and Liu Jianyun. Effect of Qianlieshu capsule on experimental prostatic hyperplasia. Pharmacology and Clinics of Chinese Materia Medica. 2007; 23(5):170-172.

15. Shang Xuejun, Cai Hongcai, Song Lebin, et al. Effect of Xialiqi capsule on expression levels of PCNA and caspase-3 in rats with benign prostatic hyperplasia [J]. Chinese Journal of Andrology. 2017, 23(8):728-733.

16. HongcaiCai, Guowei. Zhang, XuejunShang, etal.The Effect of Xialiqi Capsule on Testosterone-Induced Benign Prostatic Hyperplasia in Rats[J].Evidence-BasedComplementaryandAlternativeMedicine,2018,9(13):1-10.

17. Na Yanqun, Ye Zhangqun, Sun Yinghao, et al. Guidelines for diagnosis and treatment of urological diseases in China (Wu Jieping) [M]. Beijing, People's Medical Publishing House, 2014.

18. Truls E. Bjerklund Johansen,Timothy M. Baker,Libby K. Black. Cost-effectiveness of combination therapy for treatment of benign prostatic hyperplasia: a model based on the ﬁ ndings of the Combination of Avodart and Tamsulosin trial[J]. BJU International.2011,109:731-738.

19. Konstantinos Dimitropoulos, Stavros Gravas. Fixed-dose combination therapy with dutasteride and tamsulosin in the management of benign prostatic hyperplasia[J]. Therapeutic Advances in Urology.2016,8(1)19-28.

20. Claus G. Roehrborna,Paul Siamib, Jack Barkinc,etal. The Effects of Combination Therapy with Dutasteride and Tamsulosinon Clinical Outcomesin Menwith Symptomatic Benign Prostatic Hyperplasia: 4-Year Results from the CombAT Study[J]. European Association of Urology.2010,57:123-131.

21. McConnell JD , Roehrborn CG , Bautista OM ,etal. The long-term effect of doxazosin, finasteride ,and combination therapy on the clinical progression of benign prostatic hyperplasia[J]. N Engl J Med, 2003 ,349: 2387-2398

22. Osama O elkelany, Ryan C Owen, edward D Kim. Combination of tadalafil and finasteride for improving the symptoms of benign prostatic hyperplasia: critical appraisal and patient focus[J]. Therapeutics and Clinical Risk Management 2015(11):507–513.

23. Lowe FC , Batista J ,Berges R, etal. Risk factors for disease progression in patients with lower urinary tract symptoms/benign prostatic hyperplasia (LUTS/BPH): a systematic analysis of expert opinion. Prostate Cancer and Prostatic Diseases , 2005(8) : 206-209.

**Appendix 1: Trial flow chart**

| **Item** | **Baseline** | **4 weeks ± 2d** | **8 weeks ± 2d** | **TUR-P surgery** | **4 weeks after TUR-P surgery** |
| --- | --- | --- | --- | --- | --- |
| **Medical history** | ○ |  |  |  |  |
| **Inclusion and exclusion criteria** | ○ |  |  |  |  |
| **Informed consent** | ○ |  |  |  |  |
| **Basic Information** | ○ |  |  |  |  |
| **Vital signs, physical examination** | ○ | ○ | ○ |  | ○ |
| **Maximum urine flow rate (Q_max_)** | ○ | ○ | ○ |  | ○ |
| **Average urine flow rate (Q_ave_)** | ○ | ○ | ○ |  | ○ |
| **Prostate volume** | ○ | ○ | ○ |  | ○ |
| **Residual urine volume (RU)** | ○ | ○ | ○ |  | ○ |
| **Routine urine test** | ○ | ○ | ○ |  | ○ |
| **Blood routine examination** | ○ | ○ | ○ |  | ○ |
| **PSA** | ○ |  |  |  |  |
| **Blood biochemistry** | ○ |  | ○ |  |  |
| **IPSS score** | ○ | ○ | ○ |  | ○ |
| **NIH-CPSI score** | ○ | ○ | ○ |  | ○ |
| **QOL quality of life score** | ○ | ○ | ○ |  | ○ |
| **IIEF-5 score** | ○ | ○ | ○ |  | ○ |
| **Past medication use** | ○ |  |  |  |  |
| **Concomitant medication** |  | ○ | ○ |  |  |
| **Drug dispensing** | ○ |  |  |  |  |
| **Drug recycle** |  |  | ○ |  |  |
| **Surgery** |  |  |  | ○ |  |
| **Pathological examination of prostate** |  |  |  | ○ |  |

**Appendix 2: Study flow diagram**

**Select patients with benign prostatic hyperplasia**

**Meet inclusion and exclusion criteria**

**Sign the Informed Consent Form**

**Clinical evaluation, routine examination of blood, urine and stool, blood biochemistry examination, other examinations, scale scoring**

**Take Xialiqi capsules, 3 capsules each time, 3 times a day, for 8 weeks**

**Take 3 placebos each time, 3 times a day, for 8 weeks**

**Obtain random number from a central random system, perform randomized, double-blinded, placebo-controlled grouping**

**Outpatient follow-up visit and examination at Week 4**

**Outpatient follow-up visit and examination at Week 8**

**Perform surgery on patients who receive the surgery**

**End visit for patients who do not receive the surgery**

**Outpatient follow-up visit and examination 4 weeks after surgery**
